# Spirituality, Organizational Gratefulness, and Well-Being Among Polish Workers

**DOI:** 10.1007/s10943-024-02036-1

**Published:** 2024-04-12

**Authors:** Marcin Wnuk

**Affiliations:** Department of Work and Organizational Psychology, Adam Mickiewicz University in Poznań, Marcin Wnuk, ul. Szamarzewskiego 89/AB, 60-568 Poznan, Poland

**Keywords:** Workplace spirituality, Organizational gratefulness, Stress at work, Life satisfaction

## Abstract

The relationship between spirituality at work and occupational and subjective well-being is not a well-recognized area of research. Many studies have indicated the beneficial effects of spiritual activities on employees’ flourishing, but the mechanisms of this influence are still not sufficiently explained. This study aimed to verify the proposed mechanisms that underlie employees’ spirituality, stress at work, and life satisfaction, and the role of gratitude toward the organization in these relationships. It was assumed that employees’ spirituality is indirectly related to stress at work via gratitude toward the organization. In turn, gratitude toward the organization is directly and indirectly related to life satisfaction through stress at work. The study encompassed 754 individuals working in different companies in Poland. In a sample of women, both spirituality dimensions were indirectly related to stress at work and life satisfaction. Among men, only the secular dimension of spirituality, such as attitude toward coworkers, was indirectly related to stress at work and life satisfaction. Gratitude toward the organization was negatively directly related to stress at work and, through this variable, indirectly positively related to life satisfaction. The benefits of employees’ spirituality for their well-being were confirmed, emphasizing a grateful attitude toward the organization as a significant factor in this relationship.

## Introduction

Some studies have indicated the profits affected by religious and spiritual involvement at work, such as prosocial attitude and ethical organizational climate (Hardy & Carlo, 2005; Li & Chow, 2015; Otaye-Ebede et al., 2020), empowerment (Paul et al., 2020), work engagement (Bickerton & Miner, 2021), organizational commitment (Rego & Pina e Cunha, 2008), intention to quit the organization (Gatling et al., 2016), job satisfaction, and effectiveness (Karakas, 2010; Sharma & Singh, 2020). Karakas (2010) presented three perspectives on the benefits of workplace spirituality: human resource, which focuses on employees’ well-being; philosophic, which centers on finding meaning and purpose at work; and interpersonal, which is related to a feeling of community and connectedness. These perspectives are related, emphasizing the significant role of work meaning and social relationships in employees’ well-being.

According to recent research, both having a meaningful and purposeful life (Aglozo et al., 2021; Jang et al., 2018; Khumalo et al., 2014; Krok, 2015; Wnuk and Marcinkowski, 2014; Yoon et al., 2015) and having social support (Fatima et al., 2018; Holt et al., 2013; Leyva et al., 2015; Pirutinsky et al., 2011) as a consequence of religious and/or spiritual engagement are essential factors for well-being and mental health. Also, in the paradigm of positive psychology, researchers have tried to find factors underlying the mechanism of the relationship between spirituality/religiousness and positive outcomes by testing the roles of hope (Chang et al., 2013, 2016; Marques et al., 2013; Nell & Rothmann, 2018; Wnuk, 2023), optimism (Aglozo et al., 2021; Cheadle et al., 2018; Kvande et al., 2015; Warren et al., 2015), and moral emotions such as gratitude (Jang et al., 2018; Kane et al., 2021; Li & Chow, 2015; Szcześniak et al., 2019; Van Cappellen et al., 2016) and forgiveness (Jang et al., 2018; Kane et al., 2021; Lawler-Row, 2010; Sharma & Singh, 2020; Wnuk, 2022a). Unfortunately, these ideas are quite rarely transferred to the business area as important theoretical and practical topics that demand further exploration.

The spiritual sphere is an integral and immanent element of life that manifests in a work context. It is especially important to examine how aspects of spiritual dimension influence occupational well-being and to verify the mechanisms underlying these relationships. Despite the growing interest in spirituality at work over the last two decades, this area of research is still largely undiscovered and remains slightly mysterious, fuzzy, and disordered (Benefiel et al., 2014; Karakas, 2010). The relationship between spirituality at work and well-being is still not well-recognized and is not sufficiently known in the psychology of work. Recent research has focused on other than employees samples and global not dedicated to an occupational area measures based on non-precise, non-comprehensive, and not well theoretically grounded conceptualization of this phenomena (Hill et al., 2013).

This study aims to fill the gap in knowledge, realize the postulate of the human resources management perspective of employees’ well-being (Karakas, 2010), and respond to the question of how spirituality at work is indirectly related to well-being through gratitude toward the organization.

## Review of Literature

### Workplace Spirituality, Religiousness, and Gratitude

Poland is a very homogeneous religious country where the vast majority of believers represent the Roman Catholic Church as a relevant element that influences national identity (Pew Research Center, 2018). In colloquial convincement, it leads to identifying religiosity and spirituality and treating these as synonyms. This phenomenon is strengthened by the fact that in Poland, spiritual care, for example, hospices, medical, and care institutions, is generally provided in the context of religious support and focused on chronic and palliative patient needs (Kotlińska-Lemieszek et al., 2022) and development of competencies; the group of workers is involved in this process (Fopka-Kowalczyk et al., 2023). There is a lack of research regarding the spirituality of ordinary employees functionalizing in typical business not medical and caregiving context. Recent research indicated that religious involvement is a way of spiritual growth that is beneficial for Polish employees' well-being. For example, in the Wnuk study (2018), both prayer and mass attendance strengthened the link between relationship with God and job satisfaction.

A scientific approach to spirituality and religiosity considers religiosity and spirituality as distinct but overlapping constructs, emphasizing spirituality as more general. The common element of both of them is spiritual experiences, which can be achieved through religious beliefs and practices such as rituals, worship, prayer, or reading the Bible (Baumsteiger & Chenneville, 2015; Hardy et al., 2014; Wnuk, 2023; Yoon et al., 2015) and in a secular way outside the religious context (Galen, 2018). It means that spiritual struggles are not only the domain of religiously inclined individuals but also agnostics, atheists, and religious skeptics (Mercadante, 2020; Sedlar et al., 2018) identified as spiritual but non-religious individuals (Ammerman, 2013; Wixwat & Saucier, 2021). They represent an approach to spirituality different from the religious way, without reference to the divine and through searching for transcendence in areas other than religious.

Spirituality at work is not constructed based on one precise, universal, and theoretically well-grounded definition of this phenomenon. A plethora of definitions available in the literature represent many spirituality at work concepts (Banyhamdan et al., 2012), but they are centralized around the relationship with the divine or a “Higher Power” and attitudes toward other employees as well as meaning and functions spirituality at work (Lips-Wiersma et al., 2009; Liu & Robertson, 2011; Wnuk, 2022a, 2022b).

This study was based on the spirituality at work concept, which is broad enough to encompass dimensions of both secular spirituality and religious spirituality and does not exclude any types of employees due to worldview. It is the realization of Jurkiewicz and Giacalone’s (2004) postulate regarding a spirituality at work values framework. It means that every employee is spiritual, but for some individuals, secular values are the source of spiritual growth, while for others, the source is religion (Galen, 2018). This idea of employees’ spirituality was taken from the philosophy of self-help groups as the most open movement (fellowship), where everyone can participate and spiritually grow independently of presented secular or religious worldviews and with differences in religious affiliation (Alcoholics Anonymous, 2001).

As stated above, one dimension of employees’ spirituality is the attitude toward a “Higher Power,” which for religious individuals who are personified by God, but for religious skeptics, agnostics, and atheists, who define themselves as spiritual but non-religious, it is the perception of the “Higher Power” as a creative intelligence or spirit of the universe, etc. (Alcoholics Anonymous, 2001). Like religion, a secular worldview can be a cognitive-behavioral framework that guarantees predictability, control, coherency, direction, and meaning provided by a scientific-based philosophy, such as evolution by natural selection (Rutjens et al., 2010).

The second factor of employees’ spirituality is the attitude toward coworkers and employer, which is treated as a secular dimension of spirituality for both religious and secular workers. For representatives of the religious worldview, the antecedent of the approach to workmates is their bond with God (Wnuk, 2022b), but for individuals with a secular worldview, it can be another secular value personified by a “Higher Power”.

A relationship to a “Higher Power” is defined as a source of direction, purpose, and meaning at work, which facilitates transcendence and overcomes barriers and limits at work and is a factor of support for those struggling with daily work stress, challenging times, and crises and conflicts at work. Attitude toward coworkers and employer encompasses caring for the needs of workmates and appreciating their efforts, sharing knowledge and experience at work, accepting coworkers independent of their disadvantages and mistakes at work, and standing up against injustices at work (Wnuk, 2022b).

Gratitude has been described as a moral emotion, virtue, attitude, habit, personality trait, and coping response (Emmons et al., 2003), which has its roots in religious involvement and through humility, compassion, and finding meaning in life that leads to gratefulness to God (Krause & Hayward, 2015). Olson et al. (2019) confirmed in a 3-week longitudinal study that spiritual practices such as prayer and meditation increased gratitude. Another longitudinal study indicated that daily religiousness as a cause of daily spiritual experiences is indirectly related to growing gratitude, forgiveness, and empathy (Hardy et al., 2014). Individuals with religious involvement tend to receive more emotional support from other church members and feel a stronger spiritual connection with them, which leads to a greater gratitude toward God (Krause & Ellison, 2009). Independent of the cause-and-effect relationship between religious gratitudes measured by gratitude to God and general gratitude and the direction of this link, being grateful to God enhances the psychological benefits of gratitude accompanied by religious commitment (Rosmarin et al., 2011). Also, individuals who are not religious can shape and develop their grateful attitudes because social functioning demands that they proceed under reciprocity norms (Gouldner, 1960) as a social crucial rule serving the function of promoting relationship formation and maintenance (Algoe et al., 2008). The reciprocity norm as an obligation to repay according to a received good is the essence of social exchange (Blau, 1964) and is one of the most influential approaches for understanding workplace behavior (Cropanzano & Mitchell, 2005). Reciprocity is considered from the perspective of transactional mutual interdependent exchanges and cultural expectations that people get what they deserve (Gouldner, 1960) and moral norms (Cropanzano & Mitchell, 2005). All of these approaches take into consideration sensitivity to social learning (Bandura, 1977) and modeling a gratitude attitude in the primary and secondary socializing process (Wnuk, 2020), as well as developing in an organizational context. Recent research has confirmed that gratitude can be learned without references to the divine or religiousness (Stewart et al., 2016; Watts et al., 2006), which exemplifies many studies demonstrating that gratitude intervention leads to fruitful and beneficial effects for individuals and organizations (Cregg & Cheavens, 2021; Komase et al., 2021).

Within organizational support theory (Eisenberger et al., 1986; Rhoades & Eisenberger, 2002), social exchange occurs between subordinates and supervisors as part of a mutual exchange, where the supervisor is perceived as a personification of the organization and its representative, acting as organizational agent, and their favorable or unfavorable orientation toward an employee is assessed from the perspective of support or its lack from the organization (Levinson, 1965). Employees tend to perceive supervisor support and take care of their needs through the prism of organizational concern and repay the organization by focusing on its business goals and involvement in their realizations as a result of felt obligation based on the reciprocity norm. For example, many studies have confirmed that perceived supervisor support (PSS) indirectly through perceived organizational support (POS) is related to positive outcomes, such as job satisfaction, affective organizational commitment, performance, organizational citizenship behavior, and employee turnover (Baran et al., 2012; Eisenberger et al., 2002; Rhoades & Eisenberger, 2002). Qing et al. (2021) confirmed that employee gratitude mediates the relationship between family-supportive supervisor behaviors and work engagement.

Gratitude toward the organization is a moral attitude (virtue) consisting of the moral norm of being grateful and gratitude as a commitment to reciprocity as cognitive-emotional-motivational aspects, which are a response to the need to reciprocate (Wnuk, 2020). Due to the general stable character of the moral norm of gratitude as one dimension of organizational gratefulness reflecting the generalized conviction that one has to be and should be grateful— which is relatively independent of the reciprocity norm—the study used gratitude as a commitment to reciprocity. The main reason for this choice was to show that the relationships between human beings filling the employee role and the “Higher Power” can be subject to the same rules of social exchange and reciprocity norms as the exchange between two persons.

Research suggests that an individual’s spirituality can be analogous to a human relationship, and a secure attachment to a “Higher Power” can be a source of support, success in dealing with stress, facilitate finding direction, meaning, and purpose in life. Considering that a bond with a “Higher Power” can be an important source of support, coping, and finding work meaningful and purposeful, the employee, according to the reciprocity norm, should feel an obligation to repay. The target of this repayment can be the organization. Employees can materialize this in a grateful attitude toward the organization because they want to see themselves as grateful, repay their debts, and expect further favorable treatment. Following the anticipation of a positive approach from the organization, employees can be motivated to have a positive attitude toward workmates and employers, focusing on the search for manifestations of favorable treatment and connecting these to the need to be grateful toward the organization.

#### Hypothesis 1

Employee spirituality is positively related to gratitude toward the organization.

### Gratefulness, Stress at Work, and Well-Being

Gratitude bears the fruit of many benefits in human life, such as coping with stress, improving self-esteem, reducing materialistic striving, building social resources, motivating moral behavior, and promoting physical health (Emmons & Mishra, 2011). Gratitude interventions lead to many positive organizational effects (Komase et al., 2021). According to recent research, this moral virtue at work is connected with job performance (Grant & Wrzesniewski, 2010), work engagement (Ford et al., 2018; Kersten et al., 2021; Lee et al., 2019; Qing et al., 2021), job satisfaction (Ford et al., 2018; Lanham et al., 2012; Wnuk, 2018), counterproductive work behavior, organizational citizenship behavior (Ford et al., 2018), burnout (Kersten et al., 2021; Lanham et al., 2012), autonomy at work, job atmosphere, work-life balance, and supervisor and coworker support (Cain et al., 2019). Some studies have explained the mechanisms underlying the relationship between workplace gratitude and positive outcomes, such as physical (somatic) health (O’Connell & Killeen-Byrt, 2018), organizational citizenship behavior (Ford et al., 2018), organizational disengagement (Kersten et al., 2021), depression and anxiety (Petrocchi & Couyoumdjian, 2016), and affective organizational commitment (Wnuk, 2019). For example, organizational gratitude was indirectly related to employees’ incivility, gossip, and ostracism through self-control resources (Locklear et al., 2020).

One of the essential functions of gratitude is to facilitate coping with stress (Emmons & Mishra, 2011; Komase et al., 2021). Longitudinal studies have confirmed the direction of the relationship between these variables, indicating gratitude as a source of stress reduction and the benefit outcomes of gratitude-based intervention regarding perceived stress and the flourishing of older adults (Killen & Macaskill, 2015). Subjective well-being was related to depressive symptoms, anxiety, and stress among Australian adults diagnosed with depression and/or anxiety disorder (Southwell & Gould, 2017). Gratitude affected stress and depressive symptoms in a sample of healthcare practitioners from five hospitals (Cheng et al., 2015). Well-being, appreciation of simple pleasures, appreciation of the contribution of others, and grateful mood were affected by gratitude among representatives of the adult Dutch population (Bohlmeijer et al., 2021). Also, a meta-analysis conducted by Cregg and Cheavens (2021) has shown the efficacy of gratitude intervention in reducing depression and anxiety severity.

Research has emphasized that a lower level of stress underlies the relationship between gratitude and well-being. The role of stress-protected gratitude was explored in research by O’Connell and Killeen-Byrt (2018). These authors have shown that gratitude buffers against stress and loneliness, improving—directly and indirectly—physical health in the general population (2018). The literature appeals to some evidence that gratitude promotes adaptive coping and personal growth, filling a positive role in adjustment to daily stress events (Lau & Cheng, 2017; Taylor et al., 2022; Wood et al., 2007), and post-traumatic stress disorder (PTSD) in the case of PTSD-diagnosed individuals (Kumar et al., 2022). The relationship between gratitude toward the organization and well-being can be considered within the “broaden-and-build theory of positive emotions” explaining the benefits of gratitude to effective long-term coping with stress at work and life satisfaction through the broadening repertoire of behaviors and their flexibility, and the parallel “undoing" of the lingering effects of negative emotions, which limit flexibility and the process of broadening the spectrum of behaviors (Fredrickson, 2004).

Based on the “broaden-and-build theory of positive emotions,” and O’Connell and Killeen-Byrt (2018) study, in which stress explained the association between gratitude and physical health, some assumptions regarding the research hypothesis have been made.

#### Hypothesis 2

Gratitude toward the organization is negatively related to stress at work and positively related to life satisfaction.

Stress at work in the context of a relationship between organizational gratefulness and employees’ well-being is a research area that has not been comprehensively explored. Stress at work, in addition to job satisfaction and satisfaction with career, is one of the occupational well-being indicators, which according to the bottom-up approach to life satisfaction (Diener, 1984; Heller et al., 2004; Pavot & Diener, 2008) is related to this variable (Erdogan et al., 2012; Heller et al., 2004), the same as satisfaction from other areas of life. Life satisfaction is a cognitive part of subjective well-being regarding the overall assessment and attitudes about one’s life as a whole (Diener, 1984).

Recent longitudinal research based on the spillover hypothesis has indicated that job satisfaction and life satisfaction are associated (Unanue et al., 2017). For example, among Taiwanese high school teachers, job stress negatively explained life satisfaction (Chen, 2016). In Bell et al.’s (2012) study, job threat stress negatively predicted well-being and work-life balance and positively explained ill-being and work-life conflict.

#### Hypothesis 3

Stress at work is negatively and directly related to life satisfaction.

#### Hypothesis 4

Gratitude toward the organization can serve as a coping source that is negatively connected with stress at work and positively linked to life satisfaction, both directly and indirectly, through a lower level of stress at work.

## Materials and Methods

### Participants

Participation in the study was anonymous and voluntary. The research encompassed 754 full-time employees with employment contracts in different companies in Poland. The only inclusion criteria for participation were adulthood (at least 18 years old). The online questionnaires contain an introductory note, which informed the participants about the purpose of the study, the anonymous and voluntary nature of the study, and the right to withdraw from the study at any time without any consequences. Every participant expressed the written consent to participate in the study. All participants declared a Roman Catholic affiliation, which was the confirmation of the fact that Poles are very religiously homogeneous nation (Pew Research Center., 2018). The sample was balanced on the grounds of gender and education. Fewer than 60% were men, and more than 50% of the participants had higher education in comparison with slightly more than 40% with secondary education. The average age was slightly younger than 30 years old, and the seniority was little more than 8 years. Most of participants was relatively young but the range of results regarding the age was wide. The youngest participant was 18 years old, but the oldest was 77 years old.

The gratitude toward the organization scale (Wnuk, 2020) consists of two factors: gratitude as a moral norm and gratitude as a commitment to reciprocity. Each question was rated on a 5-point Likert scale ranging from 1 = strongly disagree to 5 = strongly agree. The gratitude toward the organization scale has a good internal consistency (Cronbach’s α was 0.91) (Wnuk, 2020). This measure has good psychometric properties. In the research, items referencing gratitude as a commitment to reciprocity were used.

The employee spirituality scale (Wnuk, 2022b) is a reliable measure with good internal consistency encompassing two factors: relationship with a “Higher Power” and attitude toward workmates and employer. The first has a religious character for religious-affiliated employees and is secular for non-believers, but the second is secular for both groups. This tool includes 20 items, ten for each factor. The internal consistency of this instrument, measured with Cronbach’s α coefficient, was 0.94 (Wnuk, 2022b). This study used a short version of five items per factor.

The Polish version of the perceived stress at work scale (Chirkowska-Smolak & Grobelny, 2016; Cohen & Janicki-Deverts, 2012) was applied. Participants responded on a 5-point Likert scale ranging from 5 = never to 1 = almost always (Cohen & Janicki-Deverts, 2012). This tool characterized good internal consistency which measured by Cronbach’s α coefficient was between 0.84 and 0.87. The five out of ten questions of this scale were applied in a study that, according to the results of factorial analysis, strongly loaded this measure (Chirkowska-Smolak & Grobelny, 2016).

Life satisfaction was verified by Cantrill Ladder. It is a simple and understandable measure for all groups of research participants. The Cantril Ladder consists of one question in which the respondent evaluates life satisfaction on a scale from 0 (minimum) to 10 (maximum), life satisfaction (Cantril, 1965). Czapiński used this scale during a 2-month interval, obtaining a reliability score of 0.76 (Czapiński, 1992).

### Research Model and Statistical Analysis

**Fig. 1:**
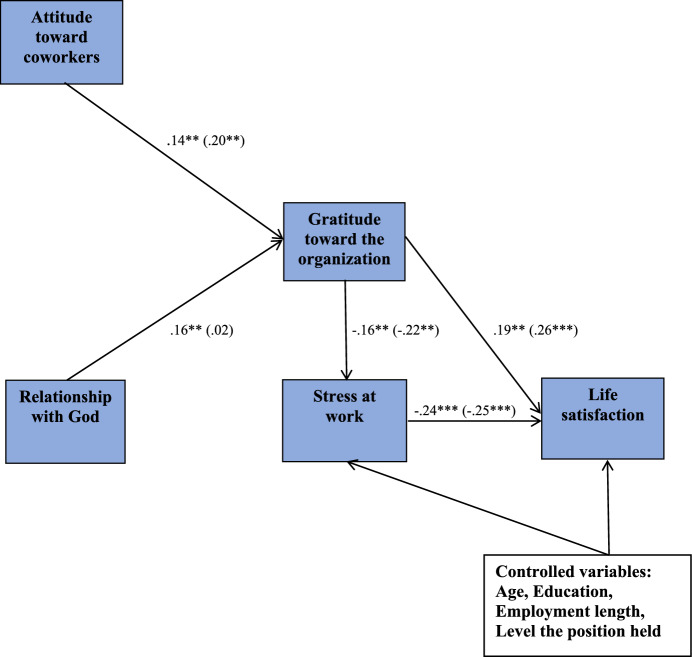
Conceptual model with results of path analysis. *Note:* The standardized regression coefficients are shown *p < .05, **p < .01, ***p < .001. Results in parentheses regarding the men but without parentheses regarding the women. The residuals for mediators were allowed to covary but are not shown for the sake of legibility. The relationship with God and the attitude toward coworkers is the dimensions of the employee spirituality scale

The prepared model (see Fig. 1) was referenced to verify gratitude toward the organization as a significant factor underlying the mechanism in the relationship between spirituality at work and occupational and cognitive aspects of subjective well-being controlled by gender, age, education, employment length, and level of position held. Also, the mediating role of occupational well-being in the relationship between organizational gratefulness and subjective well-being was tested. Spirituality at work as an independent variable was examined by a measure dedicated to employees’ spirituality, like gratitude toward the organization. Occupational well-being was tested by stress at work, but life satisfaction was applied as a cognitive indicator of subjective well-being.

Following the social exchange theory (Blau, 1964; Cropanzano & Mitchell, 2005), the function of employees’ spirituality in the exchange between employees and the organization was explored along with the role of reciprocity reflected gratitude toward the organization, which according to the “broaden-and-build theory of positive emotions” (Fredrickson, 2004), leads to well-being. Also, this theory served to expectations that a grateful attitude toward the organization is an effective way of coping with stress at work, which is indirectly through stress reduction and directly is related to life satisfaction. It was assumed that this mechanism was being strengthened thanks to social learning (Bandura, 1977) at work; therefore, the grateful attitude of workmates and supervisors is a model and an incentive for imitation. To control for the potential moderating role of gender, two separate models were tested for women and men. The literature included some implications regarding gender as a moderating variable in the relationship between gratitude and well-being (Froh et al., 2009; Hill & Allemand, 2011; Yue et al., 2017), so the potential influence of this variable needed to be verified.

The model was tested through path analysis based on the structural equation modeling (SEM) maximum likelihood method. This statistical solution was chosen because the research variable distribution was close to normal, which was verified based on skewness values that should be between − 2 and  + 2 and kurtosis values between − 7 and + 7 (Hair et al., 2010). To test whether the model was fitted to the data, arbitrary indicators were chosen as an appropriate measure of fitting, such as the root mean square error of approximation (RMSEA), Tucker-Lewis index (TLI), goodness of fit index (GFI), and comparative fit index (CFI). To evaluate the indirect effects, the bootstrapping method was used with 5,000 bootstrap resamples and 95% interval confidence. Both bias-corrected (BC) percentile methods for 95% confidence intervals were derived. When the values of upper level (UL) and lower level (LL) did not include a zero, the test statistic was significantly different from zero (Preacher & Hayes 2008). Before the model was verified, confirmatory factor analysis (CFA) was applied to compute the construct validity and discriminant validity of the research measures (Rönkkö & Cho, 2022). Additionally, Harman’s single factor test was used to exclude potential common method bias (Podsakoff et al., 2003).

## Results

Table 1 presents the socio-demographic statistics of the study sample, Table 2 presents the descriptive statistics of the variables, and Table 3 presents the CFA results.

**Table 1:** Sociodemographic characteristics of the research sample (*N* = 754). Source: (author’s research)

Variables	*N*	%
Gender		
Men	304	40.4
Women	450	59.6
Age (years; M ± SD)	29.54 (± 10.22)	
Seniority	8.19 (± 9.17)	
Educational level		
Elementary	4	0.5
Vocational	29	3.8
Secondary	312	41.4
Higher	409	54.2
Level the position held		
Ordinary workers	260	34.5
Independent specialist	319	42.3
Low mananegrs	57	7.6
Middle managers	86	11.4
Senior managers	32	4.2

*M* mean; *SD* standard deviation

**Table 2:** Descriptive statistics for the study variables (*n* = 754)

	Employee spirituality scale - relationship with god dimension	Employee spirituality scale - attitude toward coworkers dimension	Gratitude toward organization	Stress at work	Life satisfaction
Mean	16.11	27.08	28.68	13	7.09
SD	7.74	3.08	5.78	3.9	1.72
Skewness	.10	− 1.67	− .28	.29	− 1
Kurtosis	− 1.24	4.75	.01	.14	1.63
Minimum	6	6	8	5	0
Maximum	30	30	40	25	10
VIF	1.02	1.02	1.08	1.05	
Reliability	.97	.84	.90	.85	–

**Table 3:** Correlation matrix (*n* = 754)

	1	2	3	4	5	6	7	8
1. Employee spirituality scale - relationship with god dimension								
2. Employee spirituality scale - attitude toward cooworkers dimension	− 0.01							
3. Gratitude toward an organization	0.11**	0.16**						
4. Stress at work	0.07	− 0.05	− 0.22**					
5. Life satisfaction	0.11**	0.07	0.30**	− 0.30**				
6. Age	0.22**	− 0.07	0.05	0.01	0.08*			
7. Education	0.02	0.10*	0.16**	− 0.10**	0.12**	0.11**		
8. Employment length	0.19**	− 0.04	0.04	0.02	0.04	0.79**	0.10*	
9. Level the position held	0.09*	− 0.11**	− 0.17**	0.04	0.19**	0.41**	0.26**	0.33**

*p ≤ 0.05

**p ≤ 0.01

Results of the CFA confirmed good construct validity of the measures applied in the study and satisfying discriminant validity between them. According to the CFA outcome solution, four factors explained 65.28% of the model variance. Table 4 presents the values of the items loading particular factors. Life satisfaction items loaded the factor of stress at work more than − 0.3 and gratitude toward the organization at 0.25; the rest of the items theoretically assigned to a particular factor loaded this factor more than 0.65 and parallelly loaded other factors less than 0.15. The result of the life satisfaction item is not proof that it is a stress-at-work indicator because its correlation with this factor was insufficiently strong. In the literature, depending on the cutoff criterion used, the value of factor loaded should be more than 0.4 in the most liberal approach and more than 0.6 in the more restrictive one (Rojas-Valverde et al., 2020).

**Table 4:** Results of confirmatory factor analysis among items being a part of research measures (*n* = 754)

Items	Factors			
	1	2	3	4
*Perceived stress at work scale*				
In the last month, how often have you been upset because of something that happened unexpectedly at work?	− .064	.003	.055	.747
In the last month, how often have you felt that you were unable to control the important things in your occupational life?	.026	.000	.001	.823
In the last month, how often have you found that you could not cope with all the things at work that you had to do?	.060	.121	− .010	.820
In the last month, how often have you been angered because of things that happened at work that were outside of your control?	− .024	− .067	.012	.788
In the last month, how often have you felt difficulties at work were piling up so high that you could not overcome them?	.044	.008	− .035	.810
*Gratitude toward the organization scale*				
I feel grateful to my organization/company	.110	.759	− .070	− .042
I have learned a lot while working in this organization/company	− .111	.764	.143	.047
I have gained a lot of valuable experiences while working here	− .119	.717	.138	.025
I am glad that I could get to such a nice organization/company	− .056	.808	.015	− .097
I feel obliged to the organization for everything I have received from it	.119	.737	− .116	.087
I am grateful to the organization/company for the possibility to meet many valuable people	.030	.689	− .019	.043
I have received a lot while working here	− .028	.859	− .022	.060
I feel proud that I work in this organization/company	.013	.805	− .052	− .097
*Employee spirituality scale – attitude toward coworkers dimension*				
I am convinced that every employee deserves respect regardless of his or her duties	.013	− .032	.768	− .008
At work I behave in such a way as not to harm my workmates	.013	.023	.695	.018
I am glad to share my knowledge and experience at work	.028	.077	.675	.031
I have respect for every employee, regardless of the position he or she holds	− .008	− .031	.829	− .051
I am able to notice and appreciate other employees’ effort	− .006	− .004	.716	.049
I believe every employee has inalienable dignity regardless of what he or she does	.040	− .032	.793	− .023
*Employee spirituality scale – relationship with god dimension*				
In difficult moments at work I turn to my Higher Power (for example, God)	.934	.004	.033	.007
I ask my Higher Power (for example, God) for help in doing my daily duties at work	.939	.005	.013	.002
The Higher Power (for example, God) gives me hope that matters at work will move in the right direction	.937	− .022	.017	.003
My Higher Power (for example, God) is a source of comfort for me at work	.935	.007	.003	− .004
Thanks to the Higher Power (for example, God) I am able to overcome my limitations at work	.944	− .001	− .001	− .004
I am sure that my higher power (for example, God) will help me manage in difficult moments at work	.945	− .007	− .002	− .001
*Cantrill ladder*				
How is your overal satisfaction in life	.120	.251	.028	− .347

To verify common method bias, Harman’s single factor test using factor analysis was applied using a single factor solution without rotation. The Chi-square statistic of Bartlett’s test of sphericity was significant (Kaiser–Meyer–Olkin [KMO] statistic = 0.887 (df = 325); *p* < 0.001). Analysis of the principal component method revealed that the single factor solution explained 22.46% of the variance, which was significantly lower than the threshold of 40%. This means that the present study is not biased by the common method. The research hypotheses were tested with SEM using the maximum likelihood method. The choice of this method was dictated by the distribution of the study variables close to normal distribution because the values of skewness and kurtosis were under the permissible level (see Table 1).

Applied model fit indices confirmed that the model was a good fit for the data: *χ*^2^(26) = 53.21; *p* < 0.001; CMIN/df = 2.04; CFI = 0.97; GFI = 0.98; TLI = 0.92; SRMR = 0.041; RMSEA = 0.042 (90% CI [0.026, 0.059]). The values of the applied fit indicators CFI, TLI, and GFI > 0.90, RMSEA < 0.08 filled the criterion for approval to claim that the model was well-fitted (Kline, 2005). The good model fit was confirmed by the results of the Bollen-Stine bootstrapping method (*p* = 0.47).

In the sample of women, the direct effects of attitude toward coworkers and relationship with “Higher Power” on gratitude toward the organization were significant: (95% CI [0.44, 0.254], *p* = 0.006, *β* = 0.14) and (95% CI [0.58, 0.267], *p* = 0.005, *β* = 0.16), respectively. Among the men, the direct effect of attitude toward coworkers on gratitude toward the organization was significant (95% CI [0.81, 0.317], *p* = 0.002, *β* = 0.20), but the direct effect of the relationship with “Higher Power” on gratitude toward the organization was insignificant (95% CI [− 0.106, 0.143], *p* = 0.729, *β* = 0.02).

The group of women noticed statistically significant direct effects between gratitude toward the organization and stress (95% CI [− 0.273, − 0.62], *p* = 0.001, *β* = − 0.16) and between gratitude toward the organization and life satisfaction (95% CI [0.89, 0.296], *p* = 0.001, *β* = 0.19); the same as in men, which were (95% CI [− 0341, − 0.98], *p* = 0.001, *β* = − 0.22) and (95% CI [0.146, 0.378], *p* = 0.000, *β* = 0.26), respectively. Both women and men noticed statistically significant direct effects between stress and life satisfaction: (95% CI [− 0342, − 0.145], *p* = 0.000, *β* = − 0.24) and (95% CI [− 0.373, − 0.146], *p* = 0.001, *β* = − 0.25), respectively.

In the group of women, attitude toward coworkers was significantly, negatively, and indirectly related to stress at work through gratitude toward the organization (95% CI [− 0.57, − 0.7], *p* = 0.003, *β* = − 0.02) and significantly, positively, indirectly related to life satisfaction through gratitude toward the organization and gratitude toward an organization and stress at work 95% CI [0.10, 0.72], *p* = 0.004, *β* = 0.03). The same results were noticed in men: (95% CI [− 0.93, − 0.15], *p* = 0.001, *β* = − 0.04), and 95% CI [0.26, 0.120], *p* = 0.001, *β* = 0.06), respectively.

Additionally, in the group of women, a relationship with “Higher Power” was negatively and indirectly related to stress at work through gratitude toward the organization (95% CI [− 0.57, − 0.9], *p* = 0.001, *β* = − 0.04), but among men, it was not (95% CI [− 0.36, 0.24], *p* = 0.685, *β* = − 0.004). Also, among women, a relationship with God was positively and indirectly related to life satisfaction CI [0.12, 0.76], *p* = 0.002, *β* = 0.03) in comparison with the non-significant relationship in men CI [− 0.34, 0.50], *p* = 0.710, *β* = 0.007).

In both the sample from women and from men, gratitude toward an organization was positively and indirectly related to life satisfaction via stress at work: 95% CI [0.15, 0.78], *p* = 0.001, *β* = 0.04) and 95% CI [0.24, 0.107], *p* = 0.001, *β* = 0.05), respectively.

Additionally, gender was not a moderator in the connections between a relationship with a “Higher Power” and organizational gratefulness CMIN (df = 1) = 3.06; *p* = 0.080, between attitude toward workmates and organizational gratefulness CMIN (df = 1) = 0.01; *p* = 0.951, the same as the between organizational gratefulness and stress at work CMIN (df = 1) = 0.45; *p* = 0.499, as well as organizational gratefulness and life satisfaction CMIN (df = 1) = 1.73; *p* = 0.188.

## Discussion

This study explored gratitude toward an organization as a variable underlying the relationship between spirituality at work and well-being and the mediating role of stress at work in the link between gratitude toward the organization and life satisfaction. The theoretical framework for research consideration and the basis for hypothesis verification was social exchange theory (Blau, 1964), which broadens and builds on the theory of positive emotions (Fredrickson, 2004).

According to Hypothesis 1 (about positive correlations between employees’ spirituality and gratitude toward the organization), spirituality at work positively predicted organizational gratefulness attitude in reference to both spiritual dimensions but only among women. Both relationships to a “Higher Power” and attitude toward coworkers and employer positively predicted gratitude toward the organization. More intimate and supportive bonds with a “Higher Power” and more open, benevolent, accepting, indulgent treatment of other employees, and feeling unity with workmates were related to more manifestations of gratefulness focused on the organization.

The lack of a link between a relationship with a “Higher Power” and organizational gratefulness in the group of men may mean that women are more prone to reciprocity in exchange with a “Higher Power.” Still, for men, the bond with a “Higher Power” is not considered a framework to exchange with it—or they use other forms of debt repayment. This is inconsistent with some studies that have emphasized that men have a greater tendency toward reciprocal behaviors than women do (Dittrich, 2015; Thompson & Bergeron, 2017). Investment theory has shown that females demonstrate stronger dependence and stronger commitment than males in intimate relationships (Lin & Rusbult, 1995). This means that involvement in a bond with a “Higher Power” as an intimate relation can be a form of reciprocity for women but not for men. Despite the lack of correlation between the bond with a “Higher Power” and organizational gratefulness in men and the presence of this connection among women, there was no proof that gender was a moderator in this link. The lack of a moderated role of gender was also noticed in relationships of gratitude toward the organization and stress at work and gratitude toward the organization and life satisfaction (Froh et al., 2009; Hill & Allemand, 2011; Yue et al., 2017).

Due to a lack of comparable studies grounded in a business context, achieved outcomes can be confronted with studies where religious involvement enhanced gratitude to God (Krause & Ellison, 2009; Krause & Hayward, 2015; Rosmarin et al., 2011) and the global tendency to gratefulness (Hardy et al., 2014) unequivocally indicated a relationship with God as a source of a grateful behavior. The same tendency was noticed in the male group of employees. Considering the Roman Catholic affiliation of research participants, it seems reasonable to assume that for them, the “Higher Power” was identified as God, and the gratitude that they feel for him was also generalized to the organization as an expression of gratitude for all they received from him. This phenomenon was not observed in the female group of employees. It means that Polish women are appropriate targets for intervention focused on developing close, intimate, trustful, and accepting bonds with God as a way to improve gratefulness, for example, through spiritual practices like prayer or meditation. This connection with God can be an opportunity to notice how someone was gifted by god and according to the reciprocity rule can obligate to repay also in the occupational area of functioning. A recent study confirmed that in a Polish sample, prayer is beneficial for job satisfaction and amplifies the positive link between a relationship with God and job satisfaction (Wnuk, 2018).

Hypothesis 2 about the link between organizational gratefulness and stress at work and life satisfaction was fully confirmed. As expected, being grateful for the employer filled the stress-protecting function and predicted life satisfaction. This corresponds with recent studies, providing an additional argument for the significant role of this moral virtue in the stress regulation process (Cheng et al., 2015; Cregg & Cheavens, 2021; Emmons & Mishra, 2011; Killen & Macaskill, 2015; Komase et al., 2021), and the variable that is related to positive outcomes like job performance (Grant & Wrzesniewski, 2010), work engagement (Ford et al., 2018; Kersten et al., 2021; Lee et al., 2019; Qing et al., 2021), job satisfaction (Ford et al., 2018; Lanham et al., 2012), organizational citizenship behavior (Ford et al., 2018), or burnout (Kersten et al., 2021; Lanham et al., 2012). The main difference in comparison with previous research is the fact that, in earlier studies, gratefulness was used as a global tendency rather than as a variable dedicated to organization. Regardless, the same findings were made in reference to profits of gratitude as a moral virtue, which should be developed in business and taught to employees as a manifestation of corporate and individual health.

The Hypothesis 3 reference to the link between stress at work and life satisfaction was confirmed, emphasizing the harmful function of stress at work on other areas of life measured by life satisfaction. It was the next study providing evidence for the important role of occupational well-being in subjective well-being, regardless of the measure, to verify both constructs (Bell et al., 2012; Erdogan et al., 2012; Heller et al., 2004; Unanue et al., 2017).

According to Hypothesis 4, the mediating role of stress at work in the link between organizational gratefulness and life satisfaction was confirmed. Gratitude toward the organization was related to a lower level of stress, which, in turn, predicted higher life satisfaction. As in O’Connell and Killeen-Burt’s (2018) study, gratitude protected against stress and indirectly, through the lower level of stress, was related to well-being. The beneficial role of gratitude as an effective way of coping with work stress, which is indirectly related to higher life satisfaction, was confirmed. It shows that gratitude toward the organization can serve as a buffer against stress at work, protecting from harmful influence of stress, which reduces life satisfaction. It also draws attention to the potential of gratitude-focused interventions to enhance well-being which is coherent with earlier studies unequivocally indicating profits and desirable effects of gratefulness-improvement directed intervention (Cregg & Cheavens, 2021; Komase et al., 2021).

The conducted research implies some theoretical findings. The mechanism underlying the link between spirituality at work and occupational and subjective well-being was positively verified, showing that the benefits come from being grateful to the organization as a factor protecting against stress and improving life satisfaction. This idea has been explored in positive psychology but with global indicators of spirituality/religiousness, gratitude, and well-being (Jang et al., 2018; Kane et al., 2021; Li & Chow, 2015; Szcześniak et al., 2019; Van Cappellen et al., 2016). This approach was adopted on business grounds. It can be explained from the perspective of social exchange and the bond with a “Higher Power” as a mutual relationship, which demands gratefulness and reciprocity from both these parties. From the employee’s point of view, one way to repay favors, privileges, and appropriate treatment is gratefulness toward the organization and an accommodating attitude toward it. Probably stronger commitment in intimate relationships in women (Lin & Rusbult, 1995), the same as stronger spirituality (Mirkovic et al., 2021), and religiousness (Miller & Hoffmann, 1995) despite the more propensity to social exchange in men (Dittrich, 2015; Thompson & Bergeron, 2017) was the cause that this exchange had a place among women but not in men. As it was suggested due to research participants' religious inclination, it is highly probable that a “Higher Power” was identified with God and what was verified in this study, a stronger relationship with him leads to greater organizational gratefulness. Maybe for men less important role of bond with God and limited functions of this relation decide that they have other, secular values as sources of gratitude. It is possible that their stronger tendency to social exchange influences reciprocity not concerning God but in links to workmates and supervisors.

A social exchange may be suitable grounds for explaining the positive connection between attitude toward coworkers and organizational gratefulness, because the anticipation of favorable treatment and the perception of themselves as repayers of their debts can motivate them to be more grateful. Gratitude manifestations should also be consistent with the positive reception of other employees’ attitudes as favorable to counteract cognitive dissonance (Festinger, 1957).

The second important finding of this study was confirmation that stress at work mediates the relationship between gratitude toward the organization and life satisfaction, and this beneficial influence of organizational gratefulness can be considered based on broadening and building a theory of positive emotions (Fredrickson, 2004). Experiencing gratitude as one of the morally positive emotions through broadening the reactions spectrum and resources leads to more adaptability, creativity, higher stress resilience, and well-being.

The last theoretical implication refers to the need to introduce the psychology of spirituality in the education of psychologists, physicians, educators, and caregivers as a significant factor for coping with stress and the well-being of patients and clients. This area of practice should be isolated as a separate sub-discipline not necessarily related to religious issues, respecting worldviews, differences, and values possessed by clients and patients, especially those preferring the secular ones, non-religiously affiliated, with the skeptical, agnosticistic, or atheistic attitude toward the religion. Due to the religiously homogeneous nation and frequent spiritual growth through religious involvement in Poland, this problem is not as visible that in more religiously diverse societies and more secular.

Practical implications focus on giving space and permission to express spirituality at work and preparing and implementing gratefulness-centered training and workshops to prevent stress at work and improve well-being. Managers, leaders, supervisors, and human resources specialists should remember that the spiritual sphere of life is an integral part of human beings and—independent of its importance and value for particular individuals—is related to well-being at work through shaping a work attitude. Employees should be encouraged to develop gratitude on their own and have an opportunity to learn a grateful attitude in the work environment through social learning (Bandura, 1977) by using supervisors, coworkers, and other employees as models and examples. As was explored, methods stimulating gratefulness give benefits not only to individuals but also to organizations, thanks to better job performance (Grant & Wrzesniewski, 2010), stronger work engagement (Ford et al., 2018; Kersten et al., 2021; Lee et al., 2019; Qing et al., 2021), more organizational citizenship behavior (Ford et al., 2018), and less burnout (Kersten et al., 2021; Lanham et al., 2012).

### Limitations and Future Research

This study has some limitations. The result’s generalizability is limited to Polish and Roman Catholic employees from various organizations located in Poland with relatively young average ages and different positions held. Future research is expected to verify the presented model in a sample of employees with another cultural context, especially in more collective than individual cultures, and with much more diversity among religious denominations and those not religiously affiliated. Both culture and religious denomination could be potential moderators of the relationship between employees’ spirituality and organizational gratefulness. Also, other moral emotions, such as empathy and forgiveness, underlie the link between spirituality at work and well-being (Hardy et al., 2014). For a more comprehensive picture, other occupational well-being measures (not only cognitive but also emotional indicators of subjective well-being) should be applied (Diener, 1984; Heller et al., 2004; Pavot & Diener, 2008).

Lack of verification of other models is the premise for admitting that the different directions between research variables should not be excluded, even though the theoretical model was prepared based on previous studies’ results. Finally, the study’s cross-sectional (as opposed to longitudinal) design makes it impossible to present variable associations from the cause-and-effect perspective. Using path analysis allows for identifying predictor variables and outcomes variables as well as direct and indirect effects. Future research using longitudinal design should be applied to replicate the results obtained in this study and confirm this research model.

## Data Availability

The datasets used and/or analyzed during the current study available from the corresponding author on reasonable request.
